# A novel multiplex detection array revealed systemic complement activation in oral squamous cell carcinoma

**DOI:** 10.18632/oncotarget.22963

**Published:** 2017-12-06

**Authors:** Juliane Gallenkamp, Gerrit Spanier, Elisabeth Wörle, Markus Englbrecht, Michael Kirschfink, Roman Greslechner, Regine Braun, Nicole Schäfer, Richard J. Bauer, Diana Pauly

**Affiliations:** ^1^ University Hospital Regensburg, Department of Oral and Maxillofacial Surgery, Regensburg, Germany; ^2^ University Hospital Regensburg, Department of Ophthalmology, Regensburg, Germany; ^3^ Institute of Immunology, University of Heidelberg, Heidelberg, Germany; ^4^ Center for Medical Biotechnology, Department of Oral and Maxillofacial Surgery, University Hospital Regensburg, Regensburg, Germany

**Keywords:** mulitplex assay, oral squamous cell carcinoma, complement proteins, C3a, C5a

## Abstract

Oral squamous cell carcinoma (OSCC) is one of the most common tumors within the oral cavity. Early diagnosis and prognosis tools are urgently needed. This study aimed to investigate the activation of the complement system in OSCC patients as potential biomarker. Therefore, an innovative complement activation array was developed.

Characterized antibodies detecting the complement activation specific epitopes C3a, C5a and sC5b-9 along with control antibodies were implemented into a suspension bead array. Human serum from a healthy (*n* = 46) and OSCC patient (*n* = 57) cohort were used to investigate the role of complement activation in oral tumor progression. The novel multiplex assay detected C3a, C5a and sC5b-9 from a minimal sample volume of human tears, aqueous humor and blood samples. Limits of detection were 0.04 ng/mL for C3a, 0.03 ng/mL for C5a and 18.9 ng/mL for sC5b-9, respectively. Biological cut-off levels guaranteed specific detections from serum. The mean serum concentration of a healthy control cohort was 680 ng/mL C3a, 70 ng/mL C5a and 2247 ng/mL sC5b-9, respectively. The assay showed an intra-assay precision of 2.9–6.4% and an inter-assay precision of 9.2–18.2%.

Increased systemic C5a (*p* < 0.0001) and sC5b-9 (*p* = 0.01) concentrations in OSCC patients were determined using the validated multiplex complement assay. Higher C5a concentrations correlated with tumor differentiation and OSCC extension state. Systemic sC5b-9 determination provided a novel biomarker for infiltrating tumor growth and C3a levels were associated with local tumor spreading.

Our study suggests that systemic complement activation levels in OSCC patients may be useful to assess disease progression.

## INTRODUCTION

Oral squamous cell carcinoma (OSCC) is with 90% the most prevalent tumor in the head and neck area [1, 2]. It belongs to the ten most common tumors worldwide with about 300.000 cases every year and with men affected twice as often as women [3, 4]. The three most common risk factors for developing OSCC are smoking, alcohol consumption and human papillomavirus infection [3, 5]. Depending on the localization and tumor stage the therapy consists of surgical removal, irradiation and chemotherapy. In recent years, the therapeutic possibilities have been extended via targeted therapies with monoclonal antibodies like Cetuximab directed against EGF receptor or Nivolumab, a monoclonal antibody against the receptor PD-1 (programmed death) which was shown to prolong patient survival with recurring and metastasizing OSCC [6–8]. Despite these numerous therapeutic approaches and advancements, the mortality rate within the first five years is still 50%. One reason for this is missing diagnostic tools to detect high-risk lesions in early stages and differentiate between harmless and high-risk precursor lesions.

**Figure 1:**
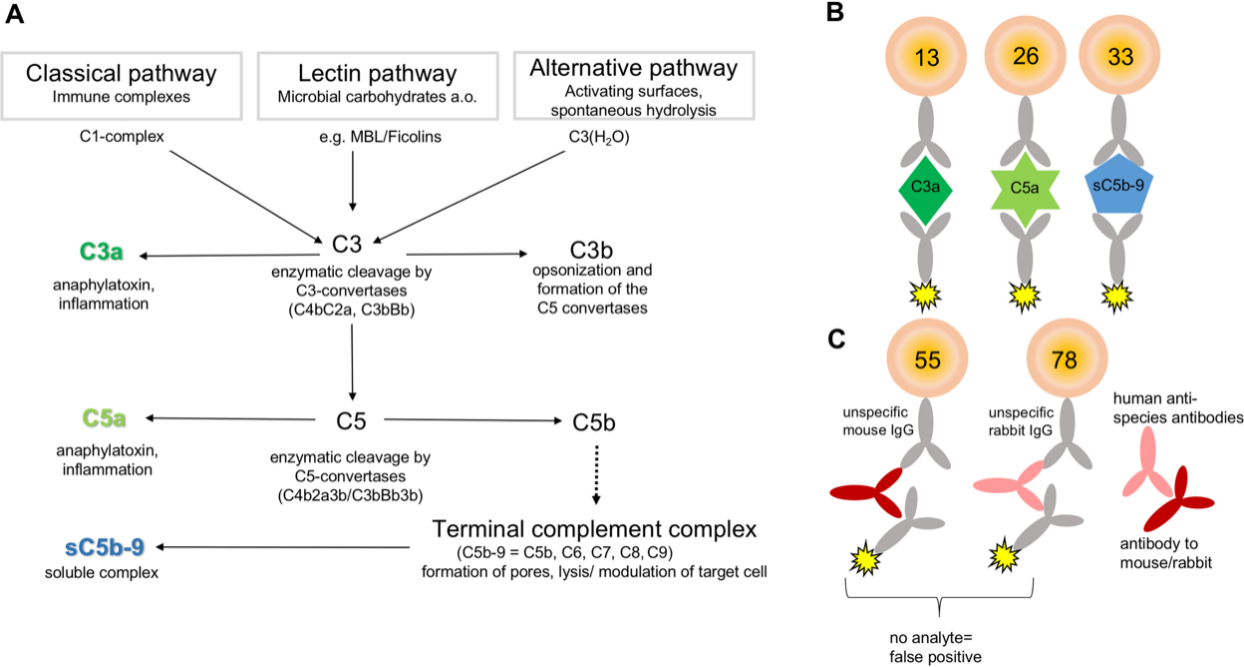
Principle for multiplex-complement activation assay (**A**) The complement system is a part of the innate immunity and activated by three different pathways: the classical, the lectin, and the alternative pathway. Activation results in enzymatic cleavage of C3 to C3a and C3b. The latter triggers the terminal complement pathway by cleavage of C5 and the formation of the terminal complement complex sC5b-C9. The formation of sC5b-C9 and the anaphylatoxins C3a and C5a determines complement activation. (**B**, **C**) The detection of the complement activation products was performed in multiplex suspension bead array based on the x-MAP technology (Luminex^®^). (B) Activation product specific antibodies were immobilized to distinct fluorescent, magnetic beads to capture either C3a, C5a or sC5b-C9 from solution, respectively. Antigen specific biotinylated antibodies and phycoerythrin conjugated to streptavidin detected the captured activation products simultaneously. (C) False positive signals by cross-reacting human anti-species antibodies and *in vitro* complement activation were excluded by utilizing an appropriate reaction buffer.

Several recent publications show complement activation via three different pathways to be involved during the development of OSCC (Figure 1A) [9]. Moreover, complement factors have been discussed as biomarkers for progression of squamous cell carcinoma [10]. High abundance of complement C3, complement factor B (CFB) and complement C4B has been demonstrated in the saliva of OSCC patients [11], especially in invasive cutaneous squamous cell carcinoma (cSCC) and recessive dystrophic epidermolysis bullosa–associated cSCCs [12]. Furthermore, the complement activation product, C5a, is involved in the development of nasopharyngeal carcinoma [13]. Other reports also reveal a role for complement proteins during ovarian and lung cancer tumor progression [14]. The precise role which complement factors play and the mechanisms by which they modulate cancer progression is still elusive.

From a diagnostic point of view, the complement system is a challenging group of proteins as the cascade is composed of more than 40 proteins, which are concerted to activated molecules and form complexes to exert their specific functions (Figure 1A) [15]. Complement activation is characterized by the generation of anaphylatoxins, C3a and C5a, which modulate and attract immune cells via their receptors C3aR and C5aR. The larger complement cleavage products, e.g. C3b, either bind to the cell surface and initiate complement activation or form the terminal complement complex (C5b-9), which integrates into the cell membrane resulting in cell lysis. If vitronectin binds to this complex in fluid-phase, the soluble terminal complement complex (sC5b-9) will be formed which is unable to integrate into the membrane remaining in plasma (Figure 1A) [16]. Most of the complement activation diagnostic is mainly performed in a single test format either by functional analysis using hemolytic assays or by enzyme linked immunosorbent assay (ELISA) detecting C3a, C5a and other activation products [17]. However, it is desirable to monitor complement activation at various levels of the cascade reaction (C3a, C5a, sC5b-9) simultaneously to verify more specifically the activation status, but also to reduce materials used and costs. Different multiplex detection platforms had been described in the past, among them the suspension bead array technology based on differentially fluorescently coded beads is of interest for a broad range of use for simultaneous immunoassays (Figure 1B, 1C) [18]. The assay principle is similar with combined sandwich ELISA: Complement activation product detecting neo-epitope specific antibodies are immobilized as capture antibodies to the color-coded beads. The bead-bound analytes are detected via biotinylated antibodies and a fluorescent reporter system. The readout is performed by a cytometer firstly addressing the fluorescent signature of the beads and secondly the intensity of the reporter signal. The current work, describes for the first time the implementation and validation of the suspension multiplex technology for a simultaneous analysis of the complement activation pattern of C3a, C5a and C5b-C9 from minimal sample volumes of human blood, aqueous humor and tear samples. Using the assay we analyzed systemic C3a, C5a and C5b-C9 concentrations as a biomarker for OSCC tumor extension and differentiation state.

## RESULTS

### Detection of complement activation using a novel multiplex suspension assay

With the growing role of the complement system in disease progression and novel complement therapeutics in clinical use (e.g. Eculizumab) and trials (e.g. Lampalizumab) complement monitoring becomes increasingly significant [19]. However, complement diagnostic is challenging because of multiple complement proteins, their interactions and cleavage products.

**Figure 2:**
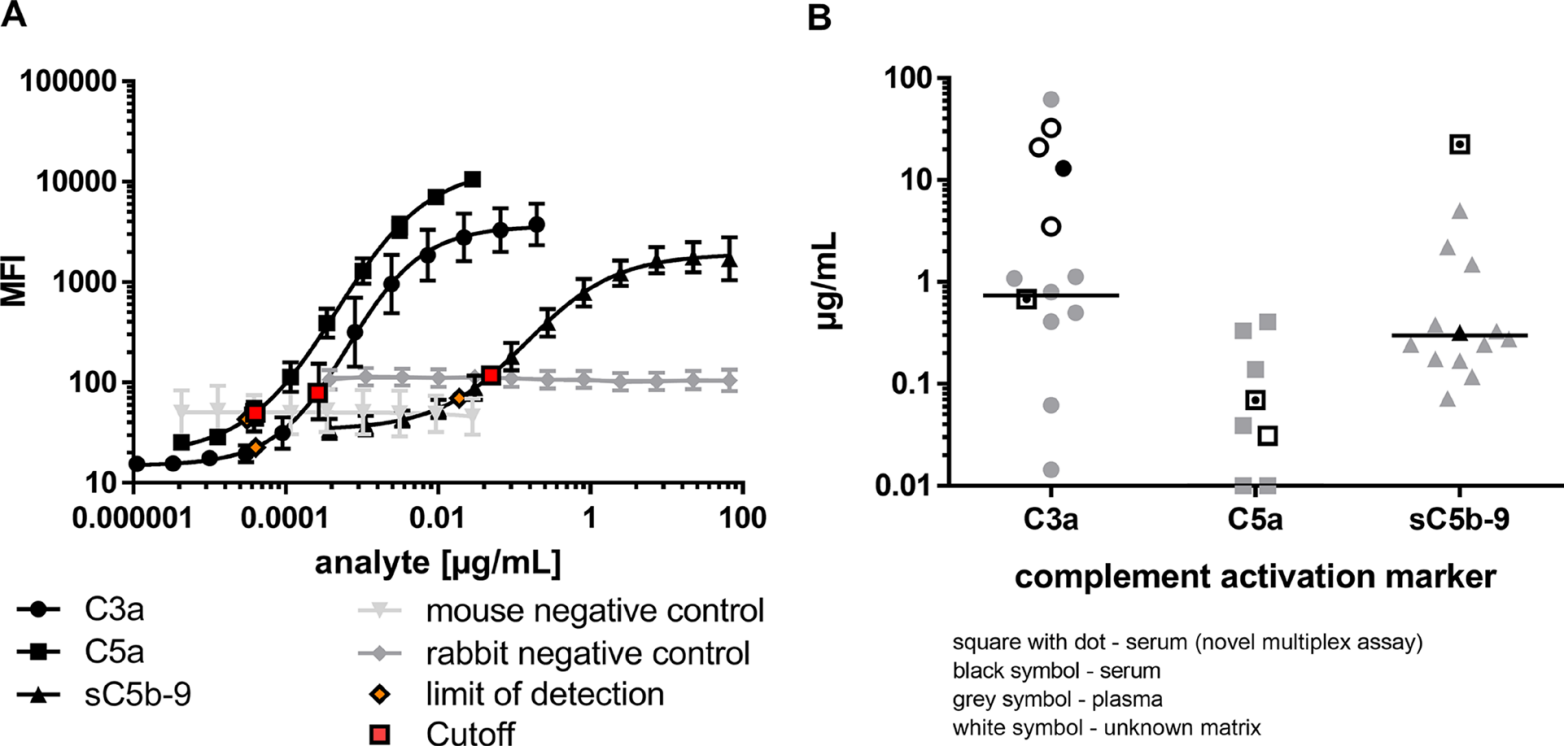
Complement activation markers were simultaneously and sensitively quantified in the multiplex system (**A**) Serial dilutions of native, human C3a-desArg (black circle), recombinant, human C5a-desArg (black square) and native human sC5b-C9 (black triangle) depicted different assay performances for the simultaneous analysis of complement activation markers. The target antigens were only detected by respective coupled magnetic beads and not by control beads (grey). Limit of detection (orange) and assay cutoffs (red square) for each analyte are shown. (**B**) The concentration range of complement activation markers determined with the novel multiplex assay from a human serum pool (black square with dot) corresponded to previously reported ranges for C3a, C5a and sC5b-C9 single detection either from plasma (grey), serum (black) or unknown sample matrix (white), respectively [42–62].

We established and validated a complement activation immunoassay by simultaneous analysis of C3a, C5a and sC5b-9 using the Luminex xMap technology and specific antibodies. The reactivity of capture and detection antibodies for the multiplex complement activation assay were evaluated in Western blots against purified complement activation products and human serum (Supplementary Figure 1). The anti-C3a capture antibody showed a binding preference for isolated C3a-fragment (Supplementary Figure 1A) and the detection antibody for the C3a containing C3 ɑ-chain in human serum (Supplementary Figure 1B). Both anti-C5a antibodies detected purified C5a in Western blots (Supplementary Figure 1C, 1D). C5a was approximately 10-times lower concentrated in human serum than C3a (Figure 2B, Figure 6). Therefore, the detection limit of the immunoblots were not sufficient for C5a analysis from human serum (Supplementary Figure 1C, 1D). The capture antibody for the sC5b-9 detected C5, C6, C7, C8, C9 and vitronectin in purified sC5b-9 complex and from human serum (Supplementary Figure 1E). The detection antibody showed a binding preference for C6 in Western blot (Supplementary Figure 1F).

**Figure 3:**
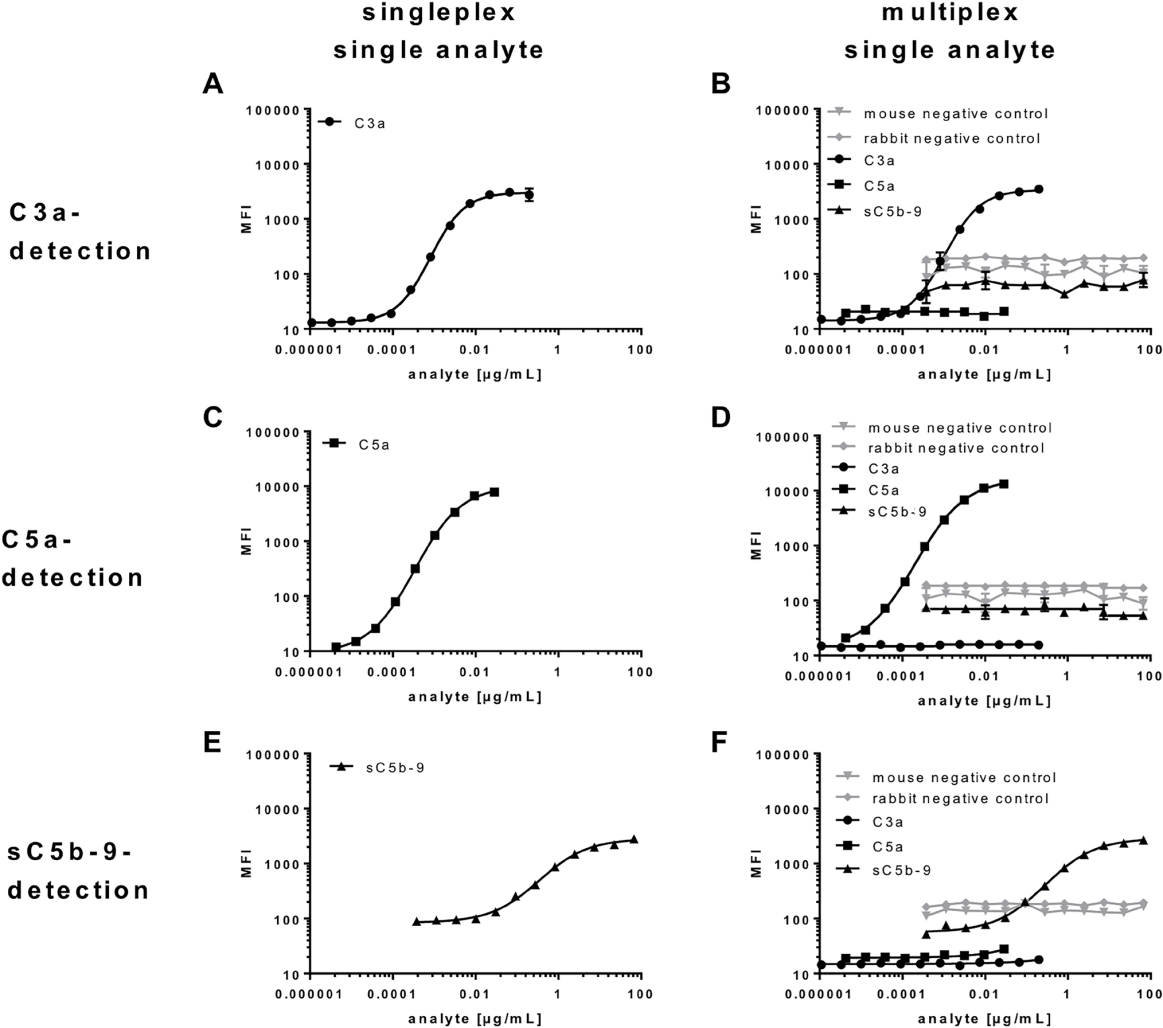
Bead-specific detection of purified antigens in singleplex and multiplex assays (**A**, **B**, **D**, **F**) Specificity of C3a-beads, (**C**, B, D, F) C5a-beads and (**E**, B, D, F) sC5b-C9-beads were either analysed in a (A, C, E) one-bead assay with a single antigen dilution series or (B, D, F) all five bead regions and a singular antigen dilution series. (B, D, F) All beads showed a specific signal for the respective antigen and no cross reactivities. The detection of (A, B) C3a, (C, D) C5a and (E, F) sC5b-C9 was comparable in the (A, C, E) single and (B, D, F) multiplex assays, indicating a bead-specific detection.

**Figure 4:**
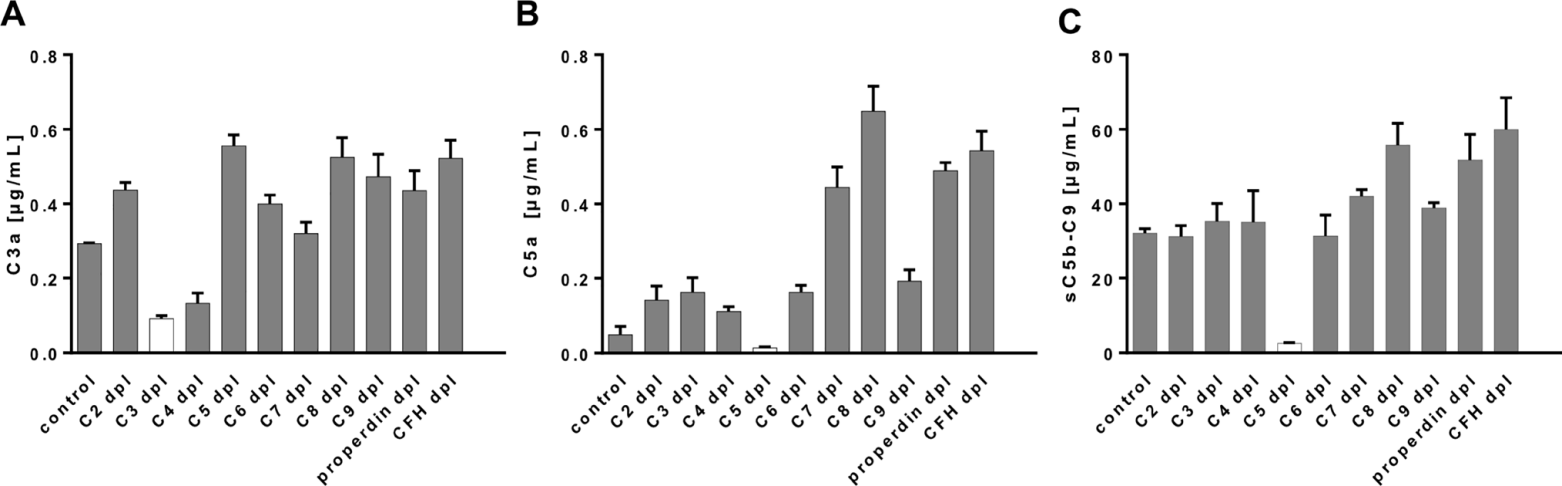
The multiplex assay detected complement activation products specifically from complement component-depleted human sera (**A–C**) C3a, C5a and sC5b-C9 were quantified either in normal human control serum (control) or C2-, C3-, C4-, C5-, C6-, C7-, C8-, C9-, properdin-, complement factor H-depleted serum. The lowest concentrations for (A) C3a and (B) C5a were observed in C3-depleted or C5-depleted serum, respectively. (C) The terminal complement complex was detected at a reduced level in C5-depleted serum. We did not observe a detection signal for any of the tested sera on the control beads (data not shown).

**Figure 5:**
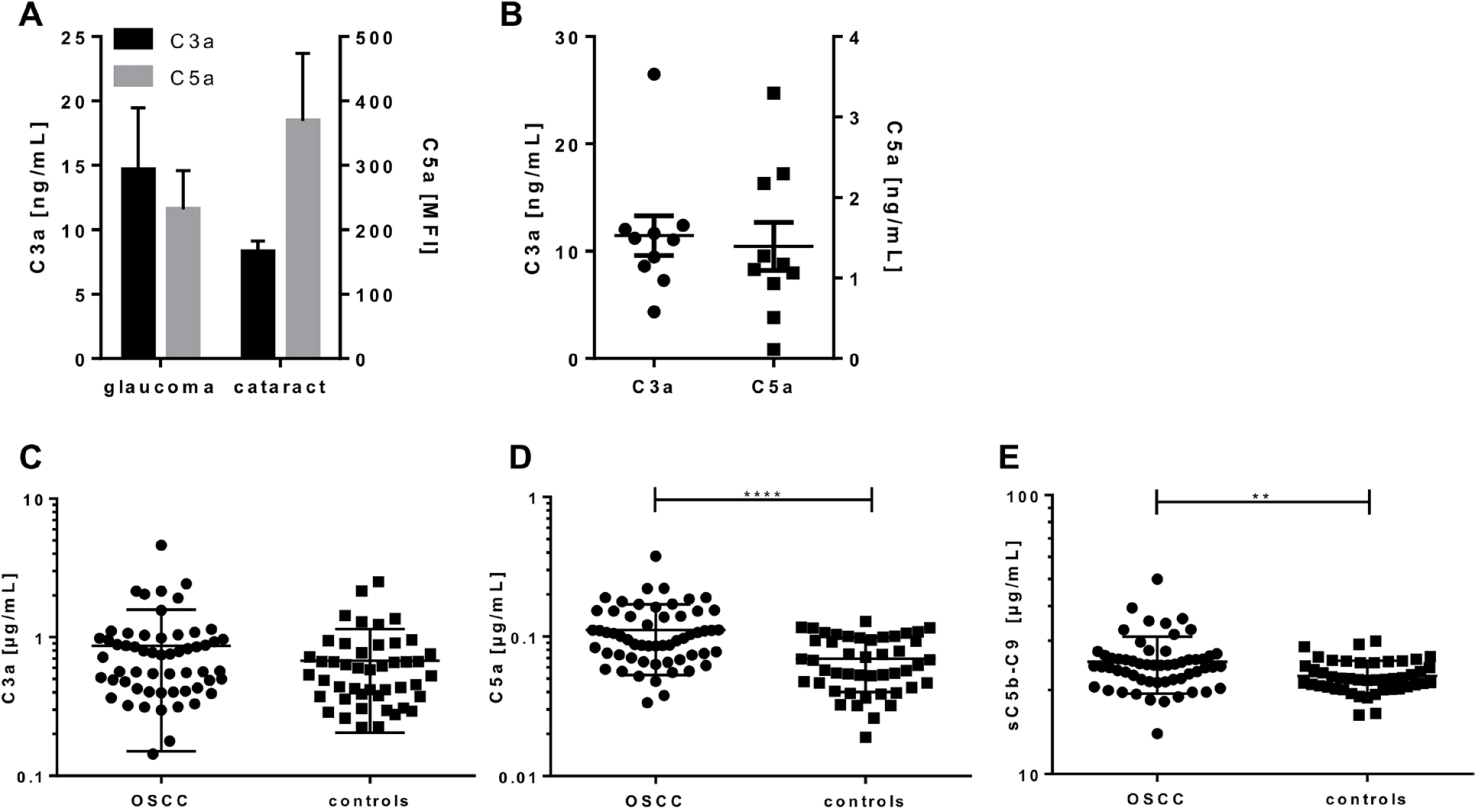
Simultaneous quantification of complement activation markers from aqueous humor, tears and sera of OSCC patients and controls C3a and C5a were either measured in (**A**) aqueous humor of glaucoma and cataract patients, (**B**) in tears of healthy controls or (**C–E**) in sera of OSCC-patients (*n* = 57) and matched controls (*n* = 46) using the validated 5-plex immunoassay based on luminex technology. (A) The C3a levels of glaucoma and cataract patients did not show any significant difference, but a tendency for higher C3a levels in glaucoma patients. C5a concentrations in aqueous humor were below the lower detection limit. (B) Quantification of C3a and C5a in tears ranged between 4.4–26 ng/mL for C3a and 0.11–3.3 ng/mL for C5a, respectively. (D, E) C5a and sC5b-C9 concentrations were significantly increased in OSCC patients compared to the control group. Mean with standard deviations are depicted. (^**^p < 0.01, ^****^p < 0.0001 two-tailed, unpaired *t*-test).

**Figure 6:**
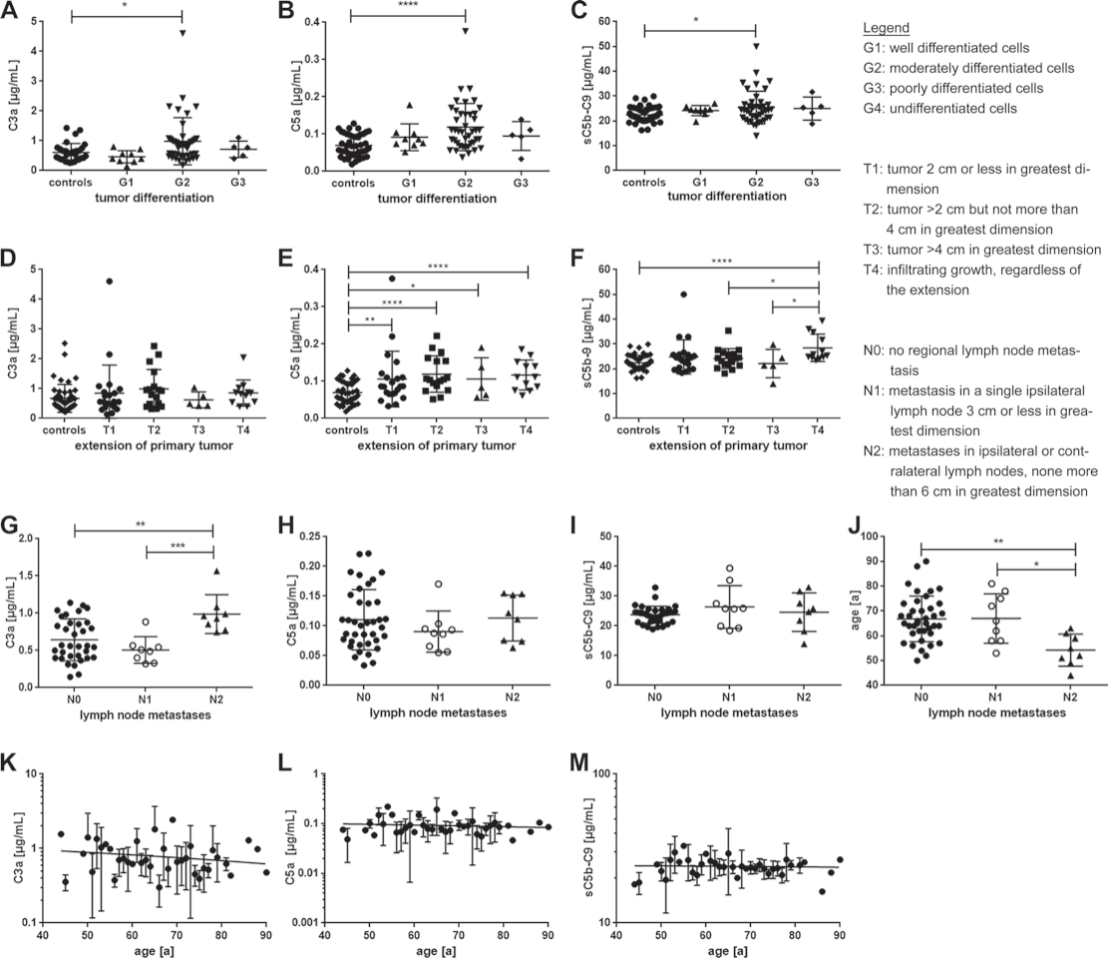
Complement activation markers were associated with tumor differentiation (G), tumor extension (T) and lymph node metastases (N) (**A–C**) The histologic grade (**G**) describes the cell differentiation in the patients’ tumors/ neoplasms. Patients with G2 tumor grading had significantly elevated (A) C3a, as well as (B) C5a and (C) sC5b-9 serum levels compared to the control group. (**D–F**) The T-classification system categorizes the extension of the patients′ primary tumors. (D) There was no relation between C3a serum levels and T-classification. (E) All tumor extension classifications (T-classifications) showed a significant increase in systemic C5a levels compared to the control group. (F) Invasive growth (T4) is associated with increased sC5b-9 serum levels compared to the control, T2 and T3 group. (**G–J**) The N-classification describes the degree of spread of tumor cells to regional lymph nodes. (G) C3a concentration was significantly correlated with tumor spread to lymph nodes. C3a concentration was highest in N2 classified lymph node metastases. (H, I) There was no significant correlation between complement markers C5a, sC5b-C9 and lymph node metastases. (J) Patients, that presented a N2-tumor, were significantly younger than patients with a N0 or N1 tumor. (**K–M**) Age was not associated with T-classification or correlated with complement activation marker concentration in serum. (^*^p < 0.05, ^**^p < 0.01, ^***^p < 0.001, ^****^p < 0.0001 two-tailed, unpaired *t*-test).

The characterized capture and detection antibodies (Supplementary Figure 1) were implemented into the novel multiplex complement activation assay in combination with the species-specific negative control antibodies (Figure 1B, 1C, Supplementary Table 1). Complement activation markers were simultaneously and sensitively quantified in the multiplex system (Figure 2A). The LOD (limit of detection) was 0.04 ng/mL for C3a detection, 0.03 ng/mL for C5a detection and 18.96 ng/mL for detection of sC5b-9 (Figure 2A, Supplementary Table 1). On average 0.68 µg/mL C3a, 0.07 µg/mL C5a and 22.47 µg/mL sC5b-9 were detected in the serum of the healthy control cohort (Figure 2B, Supplementary Table 2). The quality of the novel multiplex assay was assessed using sample material of an international inter-laboratory comparison test (complement EQA5, 2015) [19]. We determined C3a within the detection range of the reference values, however the concentrations for sC5b-9 were at factor 100 higher than the values determined by single ELISA and C5a reference levels were not provided in the round-robin test [19]. In summary, our novel assay allows a reproducible detection of C3a and C5a levels in human serum comparable to previously published studies (for references see Figure 2B), which determined complement activation markers in a comparable range using single immunoassays (Figure 2B).

Multiplex detection assays are characterized by simultaneous analysis of multiple ligands in a single tube format. Therefore, the specificity and the performance of the single and combined assays need to be validated separately to assure reliable results independently of the sample composition. Bead-specific detection of purified antigens was tested in singleplex and multiplex assays (Figure 3). The high binding capacities of the anti-C3a, anti-C5a- and anti-sC5b-9 beads were shown in a one-bead assay with a single purified antigen dilution series (Figure 3A, 3C, 3E). All three complement activation products were detected in dose-dependent logarithmic standard curves comprising four decades on a log-scale and either ranging from 0.2 to 0.00004 µg/mL for C3a (Figure 3A), 0.03–0.00003 µg/mL for C5a (Figure 3C) or 67–0.019 µg/mL for sC5b-9 (Figure 3E), respectively. The singleplex detection of each antigen was comparable with the standard curve performance in the multiplex assays, indicating a bead-specific detection (Figure 2A, Figure 3A, 3C, 3E). Moreover, unspecific cross-reactivity was tested using all five bead regions (Figure 1B, 1C, Supplementary Table 1), the three complement detecting bead regions and the two negative control bead regions, by means of singular antigen dilution series (Figure 3B, 3D, 3F). Our data showed a specific signal for the antigens with all the corresponding beads and did not reveal any cross-reactivity with unspecific bead regions or the negative control beads (Figure 3B, 3D, 3F).

Further, we determined the specificity and the cut-off levels of our multiplex assay in a more complex experimental setting using human sera depleted from specific complement factors (C2-C9, properdin and CFH) and measured the respective concentrations of C3a, C5a and sC5b-9 (Figure 4). The three complement activation products were highly specifically detected in human serum but not in the respective depleted sera (Figure 4). C3a detection was at its lowest level in the C3-depleted serum with a concentration of 0.09 µg/mL, which determined our cut-off level for the C3a detection assay (Supplementary Table 1). The remaining complement depleted sera exceeded this cut-off concentration by a factor of 1.5 (C4-depleted serum) to a factor of 6 (C5-depleted serum) (Figure 4A). The physiological serum C3a concentration in our healthy probands was at 0.68 µg/mL rising above the C3a cut off level by a factor of 7 (Figure 5C, Supplementary Table 2). C5a analysis in C5-depleted serum resulted in a cut-off determination at 0.01 µg/mL (Supplementary Table 1), which was 7-times below the physiological C5a concentration in our healthy probands and 64 times below the highest C5a concentration in C8-depleted serum (Supplementary Table 2, Figure 4B, Figure 5D). The lowest detection signal of sC5b-9 was observed in C5-depleted serum with 2.47 µg/mL. The remaining complement depleted sera showed at least 12.5 times the concentration above the cut-off (C2-depleted serum) (Figure 4C). The mean physiological sC5b-9 serum concentration in our healthy control group was at 22.47 µg/mL, which exceeds the concentration measured in C5-depleted serum by a factor of 9 (Supplementary Table 2, Figure 5E).

One major advantage of the novel complement multiplex assay is the simultaneous quantification of three activation markers from a minimal sample volume. Therefore, in a proof-of-principle, we analyzed the C3a and C5a concentrations in minimal sample volumes of human aqueous humor from glaucoma and cataract patients (<100 µL) (Figure 5A) as well as in human tears (<50 µL) (Figure 5B). The C3a concentrations in aqueous humor ranged from 1.4 to 64 ng/mL and overlapped with previously described C3a levels in the same sample matrix of healthy patients by means of single immunoassays [20]. We describe, however, for the first time very low C5a concentrations in aqueous humor samples below the cut-off levels for serum samples (10 ng/mL) using our multiplex assay (Figure 5A). C3a concentrations in tears were determined between 4.3–26.5 ng/mL, which was in accordance to previous publications (Figure 5B) [21]. A simultaneous analysis of C3a with C5a from tears has not been described before. We showed for the first time, that C5a is a component in tears at a concentration range of 1.4 ng/mL, which was below the cut-off level for serum samples but above the limit of detection (Figure 5B, Supplementary Table 1).

The variability of the 5-plex assay was determined at a dilution of 1:50 with a standard quality control serum. For each of the activation markers analyzed, the mean intra-assay precision was between 2.9%–6.4% (Supplementary Table 1). The mean inter-assay precision values for all analytes were between 9.2% and 18.2% (Supplementary Table 1). Similarly, intra- and inter-assay precision values for different cytokines in commercial multiplex cytokine xMAP-assays were reported [22, 23].

### Systemic complement activation in OSCC patients assay

C3a, C5a and sC5b-9 concentrations were quantified in 103 serum probes using the validated 5-plex assay (Supplementary Table 2). Our healthy control cohort showed a systemic complement activation concentration within a range of 0.2–2.5 µg/mL for C3a (Figure 5C), 0.02–0.12 µg/mL for C5a (Figure 5D) and 16.3–29.9 µg/mL for sC5b-9 (Figure 5E), respectively (Supplementary Table 2).

We compared the levels of C3a, C5a and sC5b-9 in serum from OSCC patient cohort (*n* = 57) to a matched healthy control group (*n* = 46) (Supplementary Table 2). Our data clearly showed a significant 60% increase of C5a (mean 0.11 µg/mL) and a 12% increase of C5b-9 (mean 25.22 µg/mL) serum concentrations in OSCC patients in contrast to the healthy control group (C5a: mean 0.07 µg/mL and sC5b-9 mean 22.47 µg/ml) (Figure 5D, 5E). However, we did not observe a significant difference in C3a concentration in serum from OSCC patients compared to healthy controls (Figure 5C).

Furthermore, correlation of our diagnostic analyses with retrospective clinical data revealed an association between the concentration of specific complement markers C3a, C5a and tumor differentiation (Figure 6A–6C) and extension (Figure 6D–6F) in serum from OSCC patients compared to healthy controls. The concentrations of all three complement activation products were significantly elevated in patients with moderately differentiated cells (G2-grade tumors) (C3a: *p* < 0.05, C5a: *p* < 0.0001, sC5b-9: *p* < 0.05), whereas there was no increase in G1 (well differentiated), G3 (poorly differentiated) and G4 (undifferentiated) tumors (Figure 6A–6C). Additionally, C5a and sC5b-9 concentrations markedly increased dependent on tumor growth comparing serum from OSCC patients and healthy controls (Figure 6E, 6F). OSCC patients with tumors sizes ranging from <2 cm to >4 cm (T1-T3) all harbored significantly higher C5a concentrations compared to healthy controls (T1: *p* < 0.005, T2: *p* < 0.0001, T3: *p* < 0.05). Moreover, patients with infiltrating tumors, regardless of the tumor size, also showed markedly higher concentrations of C5a (T4: *p* < 0.0001) (Figure 6E). Interestingly, only serum from patients with infiltrating tumors (T4) showed a significant difference in sC5b-9 concentration compared to the healthy control cohort (Figure 6F).

Furthermore, we were interested as to whether the spread of tumor cells to regional lymph nodes was associated with changes in C3a, C5a, sC5b-9 concentrations and age (Figure 6G–6J). C3a concentration clearly correlated with tumor cell spread to local metastases (Figure 6G). Metastases in ipsilateral or contralateral lymph nodes (<6 cm, N2) revealed increased systemic concentration of C3a (1.0 ± 0.26 µg/mL) compared to smaller metastases in ipsilateral lymph nodes (<3 cm, N1) (0.7 ± 0.54 µg/mL) and no metastasis (N0) (0.9 ± 0.81 µg/mL) (Figure 6G). We did not find any correlation between C5a or sC5b-9 and lymph node infiltration in serum samples from OSCC patients (Figure 6H, 6I). Notably, statistical analysis revealed a strong association between the age of OSCC patients and the lymph node spread (Figure 6J). Patients with more severe metastasis <6 cm in ipsi- and contralateral lymph nodes were on average younger (54 ± 6.4 years), compared to patients with less severe lymph node spread <3 cm and only ipsilateral and/or to patients exposing no regional lymph nodes (67 ± 9 years, *p* < 0.05 and *p* < 0.01 respectively). However, age did not correlate with C3a, C5a or sC5b-9 concentration in serum (Figure 6K–6M).

## DISCUSSION

The role of the complement system in cancer development and progression is a two-sided sword, which has been discussed in detail in recent reviews [24–27]. On the one hand, there is the anti-tumor activity of the complement system mediated by its ability to distinguish self from non-self and removing neo-antigen exposing cells or killing cells by the membrane-attack complex. On the other hand, C5a promotes tumor survival e.g. by increasing the infiltration of myeloid-derived suppressor cells and reducing the number of CD8^+^ cytotoxic T cells in the microenvironment of the tumor [28]. For further evaluation of the dual role of complement and identification of novel biomarker in tumor development and progression an improvement of complement diagnostic tools was needed to cover the multiple functions of the complement system.

We developed a bead array, which is for the first time capable of simultaneous detection of three complement activation products not only from a minimal buffer volume, but also from human serum, aqueous humor and tear fluid. While a variety of complement assays work well for single component analysis, many diagnostic tests fail when small sample volumes are tested for multiple complement proteins. Using the multiplex complement assay, we detected C3a and C5a in a comparable range as previously described single assays (for references see Figure 2B) with a high reproducibility. The use of the novel detection method provides a step towards standardized array based economized complement diagnostic as a tool for disease diagnosis and monitoring of complement-targeted therapy [29].

Using our well-validated complement activation assay we found a clear evidence of systemic complement activation in OSCC patients, as the C5a concentration was significantly elevated in OSCC sera compared to controls. Furthermore, C3a as well as C5a serum concentrations were associated with tumor differentiation, growth and extension.

It has not yet been fully elucidated, whether complement plays a disease promoting role in OSCC. However, our results indicate, that the complement system appears to be dysregulated in this cancer. The data imply, that a new therapeutic option for OSCC patients could be complement-targeting therapeutics, which modulate activation or deposition of complement factors and influence the tumor microenvironment as well as tumorigenic potential [24].

It has been discussed before that the chronicity of the inflammation affects the role of complement in the tumorigenesis, apoptosis, tumor cellular invasion and exerts a dual role on tumor growth depending on the character of immune response [25, 30]. To evade antibody mediated complement dependent cytotoxicity tumor cells can adopt diverse strategies. One strategy for OSCC cells and other solid tumors like breast and pancreatic tumors is to overexpress complement restriction factors (CD46, CD55, CD59) in different densities [31]. Gelderman *et al.* reported the deposition of C3 and C5b-9 on cervical carcinoma cells and the surrounding stroma. They demonstrated that CD55 and CD46 were the most potent inhibitors of C3 deposition. Moreover, they increased complement activation applying specific antibodies against these restriction factors and the antibodies could be immunotherapeutic agents to enhance the inflammatory reaction at the tumor site [32]. Maehara *et al.* described a mechanism for preventing hepatocellular tumor development where AIM (apoptosis inhibitor of macrophage) accumulate on the HCC (hepatocellular carcinoma) cell surface and activate the complement cascade thus inducing necrotic cell death in AIM bound HCC cells [33].

However, there is increasing evidence demonstrating that several complement factors are produced by tumor cells themselves exerting diverse tumor promoting autocrine effects. In cSCC (cutaneous squamous cell carcinoma) complement factor H and factor H-like protein-1 have been identified as potential progression markers as their gene expression was upregulated in cSCC cell lines and primary cSCC tumors [34]. In patients with ovarian and lung cancer autocrine effects of C5aR and C3aR facilitate cell proliferation via PI3K/Akt [14]. Silencing PI3K/Akt in cancer cells eliminated C5aR and C3aR effects in cancer cells. Furthermore, in ovarian and lung cancer higher tumoral C3 and/or C5aR mRNA expression were associated with decreased overall survival. Cho *et al.* demonstrated complement activation in ovarian cancer cells due to an autocrine effect which produces complement C3 protein. This process leads to alterations in the tumor microenvironment by increasing the number of myeloid-derived suppressor cells and reducing the number of cytotoxic T cells that infiltrate into the tumor, which promotes tumor growth [14]. These results are supported by data from Abbriti *et al.* who analyzed data of the current literature of potential meningioma biomarkers applying bioinformatic methods [35]. Their approach revealed complement factor C3, among 8 other proteins, to be particularly related to tumorigenesis of meningioma. In addition to that Chen *et al.* identified among other factors complement C3c (C3) to be differentially expressed in serum blood samples from 25 OSCC patients compared to 25 healthy controls via MALDI-TOF mass spectrometry [36]. Chemically induced mouse cSCC and 8 cSCC cell lines showed significant upregulation of C3 and CFB (complement factor B) gene and protein expression compared to benign papilloma and normal human epidermal keratinocytes. A knockdown of C3 and CFB expression inhibited migration and proliferation of cSCC cells and resulted in inhibition of ERK 1/2. Moreover, knockdown of C3 and CFB markedly inhibited growth of human cSCC xenograft tumors *in vivo* [12]. Jiang *et al.* found significantly higher serum levels of complement C3 and C4 in patients suffering from myeloma bone disease with more than three osteolytic lesions compared to patients with less lesions. Moreover, C4a serum levels were significantly associated with the number of osteolytic lesions. The group demonstrated that the levels of C3, C4 and C4a were highly related to the severity of bone disease. C3 and C4 serum levels could also be significantly correlated with the percentage of osteoclast precursors and the level of bone resorption metabolites (CTX and TRACP-5b) [37] Moreover, complement C3 has also been suggested to contribute to the secretion of prostaglandin E2 (PGE2) in aberrant immune responses during induced inflammation in mammals [38]. Furthermore, C5a has been reported to be generated in the cancer microenvironment. Cai *et al.* demonstrated the influence of the C5a-C5aR axis on the proliferation of human nasopharyngeal carcinoma (NPC) *in vitro.* Here, C5a promoted proliferation of tNPC cells via PCAF-mediated STAT3 acetylation [13]. Nitta *et al.* suggest a C5a release from C5 by a cancer cell membrane-bound serine protease and thus an autocrine activation of C5aR-expressing cancer cells (bile duct, colon and cholangiocarcinoma cell lines). They showed that this activation, besides invasiveness, enhances recruitment and induction of myeloid-derived suppressor cells and neovascularization [39, 40].

The current work describes for the first time the implementation and validation of the suspension multiplex technology for a simultaneous analysis of the complement activation pattern of C3a, C5a and sC5b-9 from minimal sample volumes of human blood, aqueous humor and tear samples. Using this highly specific and reproducible assay, we identified an increased concentration of systemic C5a concentrations as a potential biomarker for OSCC tumor extension and differentiation state. We propose multiplex complement diagnostic as a feasible tool for biomarker analysis in OSCC.

## MATERIALS AND METHODS

### Patients and clinical data

Blood samples were obtained by venipuncture from fasting participants. The study involved adult patients examined and treated for a newly diagnosed OSCC (*n* = 57) at the Department of Oral and Maxillofacial Surgery, University Hospital of Regensburg, between 2013 and 2016. All included patients had no previous treatment and underwent primary surgical resection of the oral lesion and neck dissection based on clinical and radiologic findings. The patients had no conditions, which are known to be associated with a relevant activation of the complement system (e.g. acute infections, asthma, arthritis, inflammatory bowel disease) except diabetes or due to their history of smoking or alcohol consumption. The patient data were obtained prospectively from interview of patients or review of medical records. OSCC were staged according to the 7th Edition of American Joint Committee on Cancer (AJCC) guidelines. Corresponding to the tumor patients a matched healthy control cohort (*n* = 46) was defined. Aqueous humor was obtained during eye surgery either of open-angle glaucoma (*n* = 13) or cataract (*n* = 10) patients. Tear fluid was collected from healthy controls using Schirmer strips. For protein extraction Schirmer strips were incubated in 100 mM NH_**4**_HCO_**3**_ (1 h, room temperature). Subsequently, acetone was added (1 h, –20°C). The strips were removed and the tubes centrifuged (10 min, 11000 rpm). The supernatant was discarded and the remaining protein pellet was air-dried, before being dissolved in assay buffer for quantification. The protocols were approved by the University of Regensburg Ethics Committee (GeschZ 12-101-0070, 13-101-0207, 15-101-0064, 11-101-0071, 16-299-101) and written informed consent was obtained from all participants.

### Proteins and antibodies

The monoclonal mouse antibody against human C3a/C3a-desArg (mAb clone 2991, cat. HM2074-IA) and the biotinylated monoclonal mouse antibody against human C3/C3a (mAb 474-biotin, cat. HM2073-BT) were obtained from Hycult Biotech (Beutelsbach, Germany). The anti-human C5a/C5a-desArg antibodies (C17/5, cat. 518202; G25/2-biotin, cat. 518306) were purchased from Biozol Diagnostica (Eching, Germany). The polyclonal rabbit anti-human sC5b-9 antibody (cat. Ab55811) was ordered from Abcam (Cambridge, United Kingdom). The goat anti-human C6 antibody (cat. A223) was obtained from Complement Technology (Tyler, TX, USA). Goat anti-mouse IgG horseradish peroxidase (HRP) (cat. 115035164), goat anti-rabbit IgG HRP (cat. 111035003), rabbit anti-goat IgG HRP (cat. 305035003) as well as Streptavidin-HRP (cat. 016030084) were acquired from Dianova (Hamburg, Germany).

### Western blot analysis

Purified recombinant human C3a-desArg (1 µg, HC2127), C5a-desArg (1 µg, HC2102, Hycult Biotech, Beutelsbach, Germany), sC5b-9 (1 µg/0.1 µg, A127, Complement Technology, Tyler, TX, USA) or normal human serum (20 µg, NHS) were separated under reducing and non-reducing conditions in either 10% or 15% SDS-PAGE and subsequently transferred to polyvinylidene difluoride membranes. The membranes were blocked (5% BSA/PBST, 1 h) und then incubated with respective primary antibodies in blocking buffer (2 h, room temperature). Excess primary antibody was removed by washing the membranes with PBST. Secondary peroxidase-conjugated anti-mouse, anti-goat, anti-rabbit IgG or Streptavidin-HRP were applied in PBST with 2% skim milk. HRP-signal was developed with Lumi-Light blotting substrate (Roche Diagnostics GmbH, Mannheim, Germany).

### Multiplex suspension assay

#### Bead-conjugation

Capture antibodies were coupled to MagPlex^®^ microspheres according to the two-step carbodiimide protein coupling protocol of the manufacturer (Luminex Corp., Austin, TX, USA). Anti-C3a-desArg (1.7 µg, mAb clone 2991, bead region 18), anti-C5a-desArg (25 µg, C17/5, bead region 26) and anti-sC5b-9 (75 µg, ab55811, bead region 33) were conjugated to 1.25 million beads/region (Supplementary Table 1). Unspecific species control antibodies for mouse IgG (anti-botulinum toxin B antibody, 25 µg, bead region 55 [41]) and rabbit IgG (cat. B11-034, 75 µg, bead region 78, Gentaur, Kampenhout, Belgium) were integrated as specificity controls.

#### Antibody biotinylation

The goat antibody to human C6 was biotinylated according to standard procedures using Biotin-NHS (Sigma Aldrich).

#### Multiplex-assay

The multiplex suspension assay was performed as previously described [18]. Briefly, all assays were carried out in 96-well black clear flat bottom polystyrene microtiter plates (cat.: 3603, Corning Incorporated, New York, USA) at room temperature, protected from light and shaken at 800 rpm. 2000 antibody-labelled magnetic fluorescent microspheres per bead region were incubated with 50 µl sample in reaction buffer (human anti-mouse antibody diluent [cat. ab19396, Abcam, Cambridge, United Kingdom], 0.1 mg/mL Nafamostat Mesilate, 0.01 M EDTA) to inhibit *in vitro* complement activation and avoid anti-species interactions (2 h). A mixture of serial 1:3 diluted human native C3a-desArg (197.5–0.001 ng/mL, cat. HC2127, Hycult Biotech, Beutelsbach, Germany), human recombinant C5a-desArg (28.1–0.004 ng/mL, Hycult Biotech, Beutelsbach, Germany) and human native sC5b-9 (66.6–0.0004 µg/mL, cat. A127, Complement Technology, Tyler, TX, USA) were used as standard curve. Human serum (1:50- 1:350), aqueous humor (concentrated) and tear proteins (0.5–8 mg/mL) were added as sample material. A cocktail of titrated biotinylated detection antibodies was incubated with the washed bead mixtures (1 h, Supplementary Table 1). Streptavidin-R-phycoerythrin (SA-PE) PJRS14 (1 µg/mL in 1% BSA/PBS, Hayward, California, USA) was added for detection (30 min). Following washing, the beads were resuspended in 125 µL 1% BSA/PBS and the fluorescent signature of the beads as well as the corresponding PE-reporter signal was recorded using the Luminex Magpix system, in combination with the Bio-Plex Manager software (Bio-Rad Laboratories, Munich, Germany).

#### Assay validation

Limit of detection (LOD) was calculated by adding three standard deviations to the mean of the mean fluorescence intensities (MFI) of sixteen blanks. The lower and the upper plateaus as well as the half maximal concentrations (IC_50_) were determined from sigmoidal fitted standard curves with a variable slope (Graphpad prism). To assess within- (intra) and between- (inter) run precision, we analysed the quality control (standardized NHS 1:50) in the linear concentration range 6-times on the same plate and same day (intra) or on different plates and different days (inter). We calculated the coefficient of variation = [(standard deviation/mean) * 100)]. Specificity was determined by measuring cross-reactivity to complement factor depleted human sera (1:50, Complement technology, Tyler, TX, USA). The multiplex assay cutoffs were determined as complement concentration in the respective complement depleted sera.

## SUPPLEMENTARY MATERIALS FIGURE AND TABLES


