# Supplementary material for: A novel multiplex detection array revealed systemic complement activation in oral squamous cell carcinoma
Source: Oncotarget. 2017 Dec 6;9(3):3001–13. doi: 10.18632/oncotarget.22963 (PMC5790441; doi:10.18632/oncotarget.22963)
Supplement: Supplementary file 1 [file oncotarget-09-3001-s001.pdf]

A novel multiplex detection array revealed systemic complement activation in oral squamous cell carcinoma

SUPPLEMENTARY MATERIALS

REFERENCES

1. Morgan BP. The Complement System: An Overview. In Complement Methods and Protocols edited by P. B. Morgan, Method in molecular Biology, Humana Press. 2000; 150:1–13.
2. Bokisch VA, Dierich MP, Muller-Eberhard HJ. Third component of complement (C3): structural properties in relation to functions. Proc Natl Acad Sci U S A. 1975; 72:1989–93.
3. Turner N, Nolasco L, Nolasco J, Sartain S, Moake J. Thrombotic Microangiopathies and the Linkage between von Willebrand Factor and the Alternative Complement Pathway. Semin Thromb Hemost. 2014; 40:544–50.
4. Preissner KT, Seiffert D. Role of Vitronectin and Its Receptors in Haemostasis and Vascular Remodeling. Thromb Res. 1998; 89:1–21.
5. Muller-Eberhard HJ. The Membrane Attack Complex of Complement. Annu Rev Immunol. 1986; 4:503.

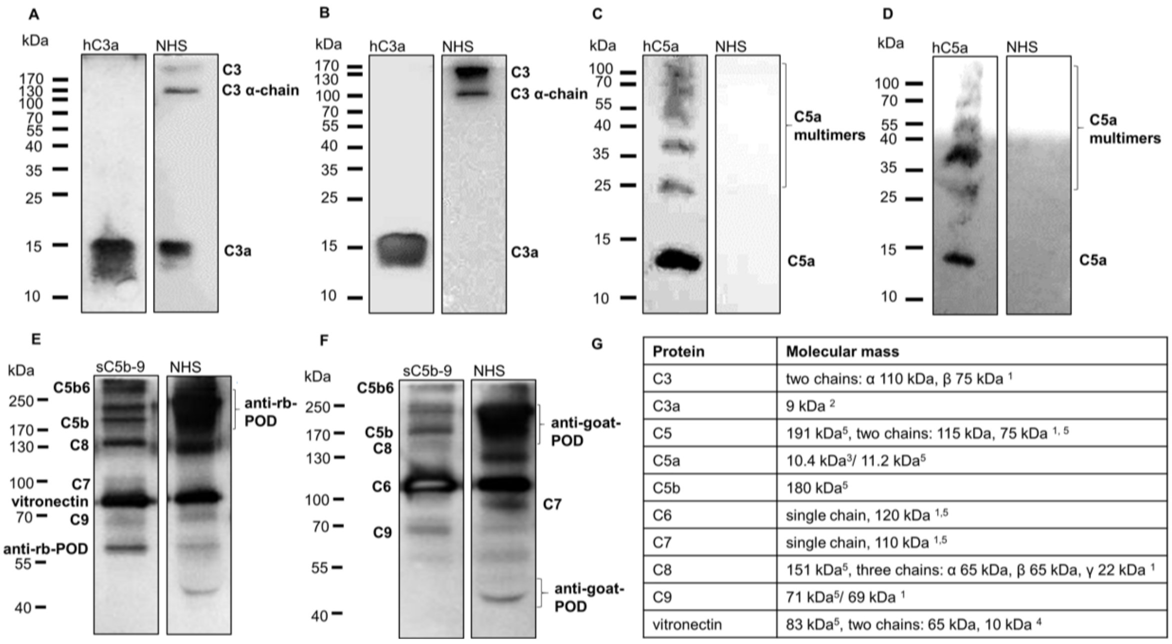

Protein	Molecular mass
C3	two chains: α 110 kDa, β 75 kDa [1]
C3a	9 kDa [2]
C5	191 kDa[5], two chains: 115 kDa, 75 kDa [1, 5]
C5a	10.4 kDa[3]/ 11.2 kDa[5]
C5b	180 kDa[5]
C6	single chain, 120 kDa [1,5]
C7	single chain, 110 kDa [1,5]
C8	151 kDa[5], three chains: α 65 kDa, β 65 kDa, γ 22 kDa [1]
C9	71 kDa[5]/ 69 kDa [1]
vitronectin	83 kDa[5], two chains: 65 kDa, 10 kDa [4]

Supplementary Figure 1: Capture and detection antibodies for the multiplex assay detected human complement proteins. (**A–F**) Normal human serum (NHS), (A, B) purified human C3a-desArg, (C, D) human C5a-desArg or (E, F) sC5b-9 were separated under non-reducing conditions either in a (A, B, C, D) 15% or (E, F) 10% SDS-PAGE. Western blot detection was performed using the corresponding antibodies for the multiplex assay (A) capturing C3a/C3a-desArg (mAb 2991), (B) detecting C3/C3a (mAb 474-biotin), (C) capturing C5a/C5a-desArg (C17/5), (D) detecting C5a/C5a-desArg/C5 (G25/2-biotin), (E) capturing sC5b-C9 (Ab55811) and (F) detecting C6 (A223-biotin). (G) Corresponding molecular weights for complement proteins are listed [[1]Morgan PB 2000; [2]Bokisch VA *et al*. 1975; [3]Turner N *et al*. 2014; [4]Preissner KT *et al*. 1998; [5]Müller-Eberhard HJ 1986].

Supplementary Table 1: Main characteristics of finalized multiplex complement activation marker assay

	Detection of C3a	Detection of C5a	Detection of sC5b-9
Bead region	13	26	33
Capture antibody	mouse anti-C3a<br>mAb clone 2991<br>3.4 µg/mL	mouse anti-C5a<br>C17/5<br>50 µg/mL	rabbit anti-C5b-9<br>Ab55811<br>150 µg/mL
Detection antibody	mouse anti-C3a<br>mAb 474-biotin<br>2 µg/mL	mouse anti-C5a<br>G25/2-biotin<br>2 µg/mL	goat anti-C6<br>A223-biotin<br>1 µg/mL
LOD	0.04 ng/mL	0.03 ng/mL	18.96 ng/mL
Lower plateau of the standard curve	<1.2 pg/mL<br>(15 MFI)	<0.2 pg/mL<br>(19 MFI)	<40 pg/mL<br>(34 MFI)
Upper plateau of the standard curve	>325 ng/mL<br>(3600 MFI)	>524 ng/mL<br>(14000 MFI)	>136 µg/mL<br>(1900 MFI)
IC_{50}	0.7 ng/mL	0.5 ng/mL	0.15 µg/mL
Intraassay variance	5.4%	2.9%	6.4%
Interassay variance	11.3%	9.2%	18.2%
Cut-off	0.09 µg/mL	0.01 µg/mL	2.47 µg/mL

Characteristic numbers for multiplex setup:
[1]Limit of detection (LOD) is three standard deviations above the mean fluorescence intensity of sixteen replicates of the zero standard in the assay; [2] For intraassay variance six duplicated samples were measured on the same plate in one single run. Coefficient of variation = [(standard deviation/mean)*100)]; [3] For interassay variance six runs on duplicate samples were performed on different days and plates. Coefficient of variation = [(standard deviation/mean)*100)]; [4] Cutoff was determined as complement concentration in the respective complement depleted serum.

Supplementary Table 2: OSCC and control cohort

	OSCC	controls
Total	57	46
Mean age [a]	65	67
Age range [a]	44–90	45–86
Male	40 (70%)	37 (80%)
Female	17 (30%)	9 (20%)
Diabetes*	11 (20%)	15 (33%)
Positive anamnesis smoking	41 (72%)	36 (78%)
Positive anamnesis alcohol consumption	37(65%)	35 (76%)
Mean BMI [kg/m^2]	25.93	27.79
Mean C3a [µg/mL]	0.87	0.68
Mean C5a [µg/mL]	0.11	0.07
Mean sC5b-9 [µg/mL]	25.22	22.47

*Type I and II.
